# Guardians of Immunity: Advances in Primary Immunodeficiency Disorders and Management

**DOI:** 10.7759/cureus.44865

**Published:** 2023-09-07

**Authors:** Nikhil Chowdary Peddi, Sravya Vuppalapati, Himabindu Sreenivasulu, Sudheer kumar Muppalla, Apeksha Reddy Pulliahgaru

**Affiliations:** 1 Pediatrics, Saint Barnabas Hospital Health System, New York, USA; 2 General Physician, People's Education Society (PES) Institute of Medical Sciences and Research, Kuppam, IND; 3 Pediatrics, People's Education Society (PES) Institute of Medical Sciences and Research, kuppam, IND; 4 Pediatrics, People's Education Society (PES) Institute of Medical Sciences and Research, Kuppam, IND

**Keywords:** immunity, immune system dysregulation, hematopoietic stem cell transplant, clinical immunology, primary immunodeficiency disease

## Abstract

Primary immunodeficiency disorders (PIDs) are a heterogeneous group of genetic conditions profoundly impacting immune function. The investigation spans various PID categories, offering insights into their distinct pathogenic mechanisms and clinical manifestations. Within the adaptive immune system, B-cell, T-cell, and combined immunodeficiencies are dissected, emphasizing their critical roles in orchestrating effective immune responses. In the realm of the innate immune system, focus is directed toward phagocytes and complement deficiencies, underscoring the pivotal roles of these components in initial defense against infections.

Furthermore, the review delves into disorders of immune dysregulation, encompassing hemophagocytic lymphohistiocytosis (HLH), autoimmune lymphoproliferative syndrome (ALPS), immune dysregulation, polyendocrinopathy, enteropathy, and X-linked(IPEX), and autoimmunity polyendocrinopathy candidiasis-ectodermal dystrophy(APECED), elucidating the intricate interplay between immune tolerance and autoimmunity prevention. Diagnostic strategies for PIDs are explored, highlighting advancements in genetic and molecular techniques that enable precise identification of underlying genetic mutations and alterations in immune function. We have also outlined treatment modalities for PIDs, which often entail a multidisciplinary approach involving immunoglobulin replacement, antimicrobial prophylaxis, and, in select cases, hematopoietic stem cell transplantation. Emerging therapies, including gene therapy, hold promise for targeted interventions.

In essence, this review encapsulates the complexity of PIDs, emphasizing the critical importance of early diagnosis and tailored therapeutic interventions. As research advances, a clearer understanding of these disorders emerges, fostering optimism for enhanced patient care and management in the future.

## Introduction and background

Primary immunodeficiency (PID) has been a topic of discussion for the past decade with a rising incidence in cases. Our immune system is unique and has different components which include but not limited to adaptive immunity which is mainly based on B-cells, T-cells, and their combined function. Innate immunity is mainly based on phagocytes and the complement system. Any disturbance in these functional cells can lead to a state of immunodeficiency. An evolving concept of immune dysregulation is also on the rise, which simply means that the immune cells are functioning but not in the way they are assigned to.

## Review

There are several disorders of adaptive immunity.

B-cell disorders

B-cells are pivotal players in the innate and adaptive immune systems, and any alterations in their functioning can give rise to a range of disorders [1,2]. B-cells are crucial in producing antibodies and mediating the adaptive immune response [3]. Dysfunction or alterations in B-cell functioning can lead to various disorders, including primary immunodeficiency disorder (PID) [3]. These disorders can result from molecular defects intrinsic to B-cells or interaction failures between B- and T-cells [3].

X-linked Agammaglobulinemia

X-linked agammaglobulinemia (XLA) is caused by a mutation in the gene responsible for producing Burton tyrosine kinase, resulting in the inability of B-cells to mature and differentiate properly [4]. This deficiency in B-cell development leads to impaired humoral immunity, as B-cells are responsible for producing antibodies in response to pathogens [5].

Clinical features of XLA typically include agammaglobulinemia: recurrent bacterial infections, particularly in the respiratory and gastrointestinal tract [6]. Patients may present with symptoms such as pneumonia, otitis media, sinusitis, and diarrhea [6]. The diagnosis of XLA involves multiple steps to confirm the presence of the disorder. First, a thorough clinical evaluation is performed, considering the patient's medical history, symptoms, and physical examination [7]. Recurrent bacterial infections, especially in the respiratory and gastrointestinal tracts, are common manifestations of XLA [7]. Laboratory investigations are crucial in diagnosing XLA [7]. One key finding is a deficiency in autoantibodies against complement components:antibody production, which can be identified by measuring immunoglobulin levels in the blood [7]. XLA patients typically have severely reduced levels of immunoglobulin, particularly immunoglobulin G (IgG) [7]. The main goal of XLA treatment is to replace the missing or deficient immunoglobulins and provide the patient with a functional immune system [8]. This is typically achieved through regular intravenous or subcutaneous infusions of IgG [8]. These infusions deliver a concentrated dose of antibodies to the patient, helping to prevent infections and improve overall immune function [8]. The future direction of XLA treatment involves further understanding the underlying genetic and immunological mechanisms of the disorder to develop more targeted therapies [9]. One area of interest is gene therapy, which aims to correct the underlying genetic defect in XLA patients [9]. This approach involves introducing a functional copy of the gene responsible for B-cell development into the patient's cells, allowing them to produce B-cells and immunoglobulins [9]. While gene therapy is still in the experimental stages, it holds promise for providing a long-term solution for XLA patients [9]. Another area of future research in XLA treatment is the development of novel immunomodulatory therapies [10]. These therapies aim to modulate the immune system in a way that improves immune function and prevents infections in XLA patients [10]. These treatments may involve the use of targeted immunosuppressive drugs to dampen overactive immune responses or the administration of cytokines to enhance immune cell function [10]. Additionally, advancements in stem cell therapy offer the potential for the treatment of XLA [10]. Stem cell therapy involves replacing or repairing defective immune cells with healthy stem cells that can differentiate into functional immune cells. This promising approach has shown success in preclinical studies and holds the potential to provide a curative treatment for XLA patients [10].

X-linked Immunodeficiency With Hyper Immunoglobulin M

X-linked immunodeficiency with hyper immunoglobulin M (IgM) is a rare PID characterized by a defect in the immune system's ability to produce IgM antibodies [11]. This condition primarily affects males and is caused by mutations in the CD40 ligand (CD40L) gene located on the X chromosome [11]. The main diagnostic criteria for X-linked immunodeficiency with hyper IgM include low levels of IgG and immunoglobulin A (IgA) antibodies, high levels of IgM antibodies, and an impaired ability to mount an adequate immune response to infections [11]. The treatment for X-linked immunodeficiency with hyper IgM primarily involves the administration of immunoglobulin replacement therapy to replace the deficient antibodies [11]. Furthermore, specific treatment strategies may be tailored to the individual based on the severity and specific manifestations of the disorder [11].

In recent years, there have been significant advancements in our understanding and management of primary immunodeficiencies, such as X-linked immunodeficiency with hyper IgM [12]. However, there are still some important future directions that need to be explored in depth. One area of research that holds promise is the development of targeted therapies for individuals with this condition [12]. Currently, immunoglobulin replacement therapy is the mainstay of treatment, but new interventions that specifically target the underlying genetic defect could potentially provide better outcomes [12]. Another important future direction is the exploration of novel diagnostic approaches. While the current diagnostic criteria for X-linked immunodeficiency with hyper IgM are effective in identifying most cases, there are still situations where an accurate diagnosis can be challenging [12].

Selective Immunoglobulin A Deficiency

Selective IgA deficiency is a PID characterized by low or absent levels of IgA antibodies in the blood [13]. It is the most common PID, affecting approximately 1 in 600 individuals. This condition can be inherited in an autosomal dominant or autosomal recessive pattern, meaning that both males and females can be affected [13]. The symptoms of selective IgA deficiency can vary widely among individuals, ranging from no symptoms at all to recurrent respiratory and gastrointestinal infections, allergies, autoimmune disorders, and inflammatory bowel disease [14]. The diagnosis of selective IgA deficiency is typically made through blood tests that measure the levels of IgA antibodies. In recent years, there have been significant advancements in the diagnosis and treatment of selective IgA deficiency [14].

Recent advancements in the field of selective IgA deficiency have shown promising developments in both diagnostic and therapeutic approaches [15,16]. One significant advancement is the use of genetic testing to identify specific genetic mutations associated with this condition [15,16]. Researchers have identified several genes, such as TNFRSF13B, ICOS, and TCF3, which are associated with selective IgA deficiency. By conducting genetic tests, healthcare professionals can confirm the diagnosis and gain valuable insights into the underlying cause of the deficiency [15,16]. Moreover, the development of targeted therapies for individuals with selective IgA deficiency is an area of active research. Currently, immunoglobulin replacement therapy is the mainstay of treatment [15,16].

Selective Immunoglobulin M Deficiency

Selective IgM deficiency is a relatively rare primary immune deficiency characterized by low or absent levels of IgM antibodies in the blood [17]. Although it is less common than selective IgA deficiency, the diagnosis of selective IgM deficiency can also present challenges [17]. The diagnostic criteria for selective IgM deficiency involve measuring the levels of IgM antibodies in the blood and excluding other specific diagnoses. However, accurate diagnosis can be difficult due to the overlap of symptoms with other immune disorders and the variability of IgM levels in individuals [17].

In recent years, there have been advancements in diagnostic approaches for selective IgM deficiency. One approach is the use of genetic testing to identify specific genetic mutations associated with selective IgM deficiency [18]. Researchers have identified several genes, such as TNFRSF13B, CD19, and CD81, that are associated with selective IgM deficiency [18].

Common Variable Immunodeficiency

Common variable Immunodeficiency, also known as CVID, is one of the most prevalent types of primary immunodeficiencies [19]. It is characterized by a reduction in the production or function of antibodies, leading to recurrent and severe infections. Unlike other forms of primary immune deficiency, which can be diagnosed through genetic testing, CVID diagnosis relies on clinical criteria and the exclusion of other specific diagnoses [19]. Individuals with CVID often present with a wide range of symptoms including respiratory tract infections (such as pneumonia and bronchitis), gastrointestinal issues (such as chronic diarrhea), autoimmune disorders, and an increased risk for certain types of cancer [20].

Due to its diverse clinical presentation and heterogeneity in terms of underlying causes, diagnosing CVID can be challenging. Measurement of serum levels of immunoglobulins, particularly IgA and IgG, is crucial in the diagnostic process [20]. In CVID, there is a severe reduction in serum levels of IgG and IgA, with normal or low numbers of B-cells. Assessing functional antibody responses can also provide valuable information in CVID diagnosis [20]. Treatment for CVID is aimed at managing the symptoms, preventing infections, and improving the immune response [21]. The management of CVID often involves a multidisciplinary approach with the involvement of immunologists, pulmonologists, gastroenterologists, and other specialists [21]. The cornerstone of CVID treatment is immunoglobulin replacement therapy, which involves administering intravenous or subcutaneous immunoglobulin to boost the levels of IgG in the bloodstream [21]. This helps to prevent infections and reduce the frequency and severity of respiratory and gastrointestinal symptoms. 

In recent years, there have been significant advancements in the diagnostic approaches for CVID. One area of advancement is the use of genetic testing to identify specific genetic mutations associated with CVID [20,22]. Researchers have identified several genes, including TNFRSF13B, CD19, and CD81, that are linked to CVID. This genetic information can provide valuable insights into the underlying causes of CVID and help guide treatment decisions [20,22]. Another important aspect of CVID diagnosis is the assessment of T-cell abnormalities [20,22,23]. T-cell abnormalities are observed in about half of CVID cases and may contribute to the defective antibody production and clinical manifestations seen in these patients [20,22,23]. Furthermore, advancements in flow cytometry techniques have allowed for more precise characterization of B-cell subsets and memory B-cell populations in individuals with CVID [20,22,23]. These advancements have improved our understanding of the immunological defects in CVID and have the potential to enhance the accuracy of diagnosis.

Hyper Immunoglobulin E Syndrome

Hyper immunoglobulin E (IgE) syndrome (HIES), also known as Job syndrome, is a rare PID characterized by recurrent bacterial infections, eczema, and elevated levels of IgE in the blood [24]. It is primarily caused by mutations in the STAT3 gene [24]. The diagnosis of HIES is based on clinical findings, such as recurrent infections (particularly of the skin and lungs), eczema, and high levels of serum IgE, along with genetic testing to identify mutations in the STAT3 gene [24]. However, it can sometimes be challenging to diagnose HIES due to the variability in presentation and the overlap of symptoms with other conditions [24].

Treatment for HIES primarily focuses on managing and preventing infections, as well as addressing the associated symptoms. The approach typically involves a combination of medications and supportive measures. Antibiotics are commonly prescribed to treat and prevent bacterial infections [25]. Prophylactic antibiotics, such as trimethoprim-sulfamethoxazole, may be recommended to prevent recurrent infections [25]. Antifungal medications may be necessary for fungal infections, while antiviral drugs may be used for viral infections [25]. In addition to medication, individuals with HIES may require regular monitoring and supportive care.

As scientific research progresses, we can anticipate the development of more precise and efficient treatments for HIES. Gene therapy is one area that researchers are focusing on, which involves correcting the genetic mutations responsible for HIES [25]. This approach has shown promise in treating other PIDs and may eventually provide a cure for individuals with HIES [25]. Additionally, advancements in immunomodulatory therapies hold potential as alternative treatment options for those affected by HIES. These therapies aim to regulate and harmonize the immune system, potentially reducing the frequency and severity of infections while enhancing overall immune function [25]. Targeting specific pathways involved in immune dysregulation, such as the Th17 pathway, could be a promising avenue for future therapeutic interventions aimed at managing HIES [25].

Transient Hypogammaglobulinemia of Infancy

Transient hypogammaglobulinemia of infancy (THI) is a type of primary immune deficiency characterized by a temporary deficiency in the production of immunoglobulins, particularly IgG [26]. It is most commonly observed in infants during the first year of life. The exact cause of THI is not fully understood, but it is believed to be due to a maturation delay in the normal production of immunoglobulins. This delay causes the physiological decrease in immunoglobulins that occurs after birth to extend beyond the first year of life [26]. As a result, affected infants may experience a decreased ability to fight off infections. However, THI is typically a self-limiting condition, with immunoglobulin levels returning to normal by 30-40 months of age [26].

Diagnosing transient hypogammaglobulinemia in infancy can be challenging, as there are no specific clinical features or diagnostic tests that can differentiate it from permanent immune defects in young children with recurrent infections and isolated low levels of IgG [21]. The diagnosis of transient hypogammaglobulinemia in infancy is primarily based on the exclusion of other hypogammaglobulinemia conditions and is later confirmed with the normalization of serum IgG levels [21].

The primary treatment involves monitoring and managing recurrent infections, providing appropriate immunizations and ensuring adequate nutrition. Antibiotic prophylaxis may be considered in certain cases to prevent severe infections [27]. Specific immunoglobulin replacement therapy is generally not necessary, as the immune system eventually catches up and normalizes on its own. However, regular follow-up with a healthcare provider is important to monitor the child's immune function and ensure that the condition resolves as expected [27].

Immunodeficiency With Thymoma

Immunodeficiency with thymoma is a rare condition characterized by the presence of a thymoma, a tumor of the thymus gland, and immunodeficiency [28]. There are two main types of immunodeficiency associated with thymoma: hypogammaglobulinemia and cellular immunodeficiency [28]. Hypogammaglobulinemia is characterized by low levels of antibodies, specifically B-cells, resulting in a weakened humoral immune response. This leads to an increased susceptibility to recurrent upper and lower respiratory tract infections caused by encapsulated organisms [28].

Additionally, patients with thymoma and hypogammaglobulinemia may also be at higher risk for bacterial, fungal, viral, and opportunistic infections due to both humoral and cell-mediated immune deficiencies [28,29]. On the other hand, cellular immunodeficiency in patients with thymoma is characterized by defects in cell-mediated immunity, such as CD4 lymphopenia and reduced T-cell mitogen proliferative responses [28,29]. This cellular immunodeficiency can also contribute to an increased susceptibility to various infections, including bacterial, fungal, viral, and opportunistic infections. The prognosis for patients with immunodeficiency associated with thymoma is believed to be worse compared to other types of immunodeficiency [28]. Patients with thymoma and immunodeficiency may experience more frequent and severe infections, including those caused by encapsulated organisms, which can be potentially life-threatening [28].

T-cell disorders

Primary immunodeficiencies due to T-cell disorders are characterized by abnormalities in the functioning of T-cells, which are an essential component of the immune system. These disorders can result in impaired immune responses, leading to an increased susceptibility to infections [30]. One aspect of T-cell dysfunction in these disorders involves an imbalance in the expression of certain immune-related genes. For example, studies have shown that the expression of FOXP3, a gene involved in the function of regulatory T-cells, is reduced in patients with T-cell disorders [31]. Thymus-derived regulatory T-cells (Tregs) play a crucial role in immune regulation by suppressing the activation of other T-cell subsets, such as Th17 and Th1 cells [31]. This impairment of Treg-mediated immunosuppression, coupled with the increased expression of genes associated with Th17 and Th1 cells, suggests a dysregulation of the immune response in individuals with T-cell disorders [31].

DiGeorge Syndrome

DiGeorge syndrome, also known as 22q11.2 deletion syndrome, is a rare genetic disorder that affects the development of several organs and systems in the body. It is caused by a deletion of a piece of chromosome 22, resulting in a variety of physical and developmental abnormalities [32]. One of the key features of DiGeorge syndrome is the underdevelopment or absence of the thymus, an organ responsible for the production and maturation of T-cells [32]. This leads to a deficiency in T-cell function, impairing the body's ability to mount an effective immune response [32]. As a result, individuals with DiGeorge syndrome are at a higher risk of infections and other immunological problems. The fully manifested syndrome is clinically characterized by several distinct features. First, there are noticeable facial abnormalities including low-set ears, a mouth that resembles the shape of a fish, widely spaced eyes (hypertelorism), ear pinnae with notches, an underdeveloped jaw (micrognathia), and downward slanting of the eyes [33]. Second, patients often present with hypoparathyroidism, which can lead to neonatal hypocalcemic tetany [33]. Third, congenital heart disease is commonly observed in individuals affected by this syndrome; particularly defects involving the aortic arch such as truncus arteriosus or interrupted arch, as well as Fallot's tetralogy [33].

Diagnosis relies on identifying the characteristic physical features associated with recurrent viral or fungal infections, such as candidiasis in the mouth and genital area or pneumonia caused by viruses [33]. An X-ray of the chest can often confirm an absence of thymic shadow and abnormalities in cardiac outline [33]. While a reduced number of circulating T-cells is commonly observed, the extent of immunological deficiency varies greatly and is only significant in about 20% of cases based on other criteria [33]. Lymphocyte proliferation in response to mitogens may also be diminished [33]. It is uncommon for patients with this condition to have antibody deficiencies [33]. The presence of a chromosome 22q11 lesion can be detected using fluorescent in situ hybridization [33]. However, it should be noted that there exists a wide range of clinical associations related to chromosome 22q11 lesions, and not all individuals with this deletion will necessarily experience clinically important immune deficiencies [33].

Currently, there is no specific cure for DiGeorge syndrome. The management of this condition involves addressing the symptoms and complications associated with the syndrome [34]. The treatment approach is typically multidisciplinary and may involve various medical specialists such as immunologists, cardiologists, and endocrinologists [34]. One of the main focuses of treatment is managing the immunodeficiency aspect of DiGeorge syndrome [34]. This may include administering prophylactic antibiotics to prevent frequent infections and vaccinating patients against common pathogens [34]. In some cases, immunoglobulin therapy may be recommended to provide the individual with antibodies that they may be lacking [34]. Furthermore, individuals with DiGeorge syndrome may require surgical intervention to repair any congenital heart defects present [34].

Future developments in the understanding and treatment of DiGeorge syndrome are focused on improving diagnostic methods and finding potential targeted therapies [34]. One area of research is the identification of specific genes or genetic variations that contribute to the phenotypic features of DiGeorge syndrome [34]. By understanding the molecular mechanisms underlying the syndrome, researchers hope to develop targeted therapies that can restore normal immune cell production and function [34]. This could potentially involve gene therapy approaches or the use of gene editing technologies. Another area of interest is the development of novel immunological treatments for the immunodeficiency aspect of DiGeorge syndrome [34]. This could include the use of immune-modulating drugs or the transplantation of hematopoietic stem cells to improve immune function [34].

Hyper IgM Syndrome 

Hyper-IgM syndrome, also known as HIGM syndrome, is a rare type of PID disease caused by mutations in the gene responsible for encoding CD40L [35]. This genetic defect results in defective immunoglobulin class-switch recombination (CSR), leading to deficiencies in three types of antibodies: IgG, IgA, and IgE [35]. However, levels of another antibody called IgM are either normal or elevated [35]. Six main forms of HIGM syndrome have been identified thus far. These forms can be divided into two categories based on their underlying causes [35]. The first category consists of mutations in genes encoding CD40L and/or CD40 proteins which play crucial roles in signaling between T-cells and B-cells [35]. The second category involves defects in genes responsible for intrinsic factors involved in immunoglobulin CSR and somatic hypermutation processes within B-cells [35].

HIGM syndrome due to CD40L or CD40 genes manifests as a combination of cellular and humoral immunodeficiencies. Conversely, intrinsic defects in B-cells result in pure humoral immunodeficiencies [35]. Regardless of the specific cause, all forms of HIGM syndrome share a common defect, the inability of B lymphocytes to undergo class-switching from IgM to other immunoglobulin isotypes [35]. As a consequence, individuals with HIGM syndrome are highly susceptible to infections and exhibit decreased serum levels of IgG, IgA, and IgE antibodies while having normal or elevated levels of IgM antibodies [35]. Notably, these individuals typically have normal numbers of circulating B-cells but may also present with defects in somatic hypermutation [35].

Patients with HIGM syndrome commonly experience recurrent sino-pulmonary infections within the first two years of life [36]. Some individuals may present with *Pneumocystis carinii* pneumonia during infancy, while others might not exhibit symptoms until adolescence when they develop parvoviral-induced aplastic anemia [36]. In addition to these infections, patients are also prone to *Histoplasma capsulatum *and *Cryptosporidium parvum *infections [36]. Chronic diarrhea is associated with *Cryptosporidium* infection, and biliary tree infection can lead to sclerosing cholangitis as well as the development of tumors in organs such as the liver, pancreas, or biliary tree [36]. Liver enzyme levels tend to increase over time causing cholangitis which is more prevalent among older patients with X-linked HIGM syndrome [36].

The mainstay of treatment is allogeneic hematopoietic stem cell transplantation (HSCT), which has been successful in most patients [37]. In some cases, additional organ transplantation, such as liver transplantation, may be required for the treatment of complications like liver failure [37]. Intravenous immunoglobulin therapy is commonly used to provide passive immunity and reduce the frequency and severity of infections in patients [37]. One potential avenue for treatment is gene therapy, which has shown success in the treatment of other immune deficiency diseases. However, gene therapy for X-linked HIGM syndrome presents unique challenges due to the highly regulated expression of the CD40L gene [38]. Current methods of gene transfer result in constitutive gene expression, which can lead to complications such as thymic lymphoproliferative disease and autoimmune disease [38]. To successfully implement gene therapy for X-linked HIGM, it will be necessary to transfer the CD40L gene with regulated expression to avoid adverse effects and ensure proper immune function [38,39].

Wiskott-Aldrich Syndrome 

Wiskott-Aldrich syndrome (WAS) is a complex immunodeficiency disorder that primarily affects males and is linked to the X chromosome [40]. It is characterized by various symptoms including thrombocytopenia, abnormal platelets, eczema, recurrent infections, and progressive deficiency in T- and B-cell function [40]. A notable aspect of this condition is the inability of individuals with WAS to produce sufficient antibodies against polysaccharides [40]. Moreover, patients with WAS are prone to developing autoimmune disorders and lymphomas [40].

The molecular basis of WAS is currently unknown. Recent studies have identified morphological and membrane abnormalities, as well as defects in signal transduction, in patients with WAS [40]. Scanning electron microscopy has revealed that peripheral T-cells and IL-2-dependent allospecific T-cell lines from these patients show a decrease in size and reduced density of microvillus surface projections [40]. These observations suggest an abnormal cytoarchitecture as the underlying cause [40]. Additionally, decreased expression of CD43 by T lymphocytes and gpl15 by platelets has been observed among individuals with WAS. It is hypothesized that activation of procalpain may render WAS patients more susceptible to enhanced cleavage of CD43 and gp115, thereby partially disrupting cell cytoskeleton morphology resembling characteristic features found in individuals affected by this syndrome [40]. The laboratory diagnosis of WAS involves a combination of clinical evaluation, medical history assessment, and specialized laboratory tests [41]. To make a definitive diagnosis, genetic testing is typically performed to identify mutations in the WASP gene, which is located on the short arm of the X chromosome [41]. Sequencing of the WASP gene can identify specific mutations that are associated with WAS [41]. Flow cytometry can also be used as a diagnostic tool to evaluate the expression of cell surface markers [41]. In individuals with WAS, flow cytometry may reveal decreased expression of certain markers, such as CD43 on T lymphocytes and gp115 on platelets [41]. Overall, the diagnosis of WAS relies on a combination of clinical evaluation, genetic testing, and flow cytometry analysis of cell surface markers [41]. The treatment of WAS requires a multidisciplinary approach to address the various aspects of the disease. There is currently no cure for WAS, and treatment focuses on managing the symptoms and complications associated with the condition [42]. One of the main components of treatment for WAS is the management of infections [42]. Due to the immunodeficiency present in individuals with WAS, they are more susceptible to recurrent infections [42]. Antibiotic prophylaxis and prompt treatment of infections are essential to prevent complications [42]. In some cases, individuals with WAS may require HSCT [42]. HSCT involves replacing the defective immune system with healthy stem cells from a donor [42].

Current research is dedicated to identifying novel biomarkers that can facilitate the early detection and monitoring of WAS [43]. This entails the development of more sensitive flow cytometry assays capable of assessing changes in the expression levels of cell surface markers like CD43 and gp115, which are known to be diminished among individuals with this disorder [43]. Furthermore, there is considerable interest in formulating targeted therapies specifically tailored to address the underlying genetic abnormality associated with WAS [43]. Encouraging approaches include gene therapy techniques such as introducing a functional WASP gene into patients' cells or utilizing gene editing technologies for rectifying mutations within the responsible gene implicated in this condition [43]. 

Severe combined immunodeficiency

Severe combined immunodeficiencies (SCID) encompass a collection of hereditary disorders characterized by significant impairments in the functioning of the immune system [44]. As a result, there is an absence or malfunctioning of T- and B-cells that originate from the thymus gland and bone marrow [44]. This condition affects both cellular and humoral adaptive immunity [44].

SCIDs can be classified in different ways based on the main pathways affected by the molecular defect or the specific immunologic phenotype related to the genetic defect [44]. One classification divides SCIDs into T-cell deficient but normal B-cell (T-B+) SCID and both T-cell and B-cell deficient SCID and further subclassified based on the presence or absence of natural killer (NK) cells (NK+ or NK-, respectively) [44]. This classification was traditionally used to determine where in the differentiation process there is a blockage due to gene alterations [44]. However, recent years have seen new causative gene alterations identified with distinct clinical and immunological phenotypes [44].

Although infants with SCID may initially seem healthy, they are highly susceptible to serious bacterial, viral, and fungal infections as the immunity passed on from their mothers declines [44]. Typical symptoms during the first year of life include failure to thrive, diarrhea, and oral candidiasis [44]. *Pneumocystis jirovecii* can frequently lead to a severe lung infection known as interstitial pneumopathy [44]. Additionally, maternal engraftment of lymphocytes can result in graft-versus-host disease [44]. In the absence of appropriate measures to restore their immune system function, SCID patients often succumb within the first two years of life [44]. For most individuals diagnosed with SCID, allogeneic HSCT is currently considered the only curative treatment option available [44]. Gene therapy has shown promise for treating specific forms of SCID; however, HSCT will likely remain the predominant method used for managing this condition in a majority of cases [44].

Recently, the implementation of newborn screening programs using T-cell receptor excision circles (TREC) has become a significant advancement in identifying patients with SCID or profound T-cell lymphopenia [45]. Compared to patients diagnosed based on clinical features, those identified through newborn screening or positive familial history can receive an early and accurate diagnosis within the first month of life [45]. As a result, they can undergo HSCT or gene therapy by three months of age before severe complications arise [45]. This approach has led to significantly improved outcomes for these patients. The TREC assay is employed in this newborn screening method which detects intracellular accumulation derived from the process of T-cell receptor gene splicing and rearrangement [45]. The assay effectively identifies several defects that lead to SCID or profound T-cell lymphopenia observed not only in typical SCID but also in individuals affected by other conditions such as 22q11.2DS, CHH, CHARGE syndrome, and AT [45]. However, it should be noted that one limitation of the TREC assay is its inability to identify all forms of combined immunodeficiency disorders or atypical SCID [45]. With further research advancements and complementary diagnostics approaches being developed to overcome its limitations, the identification rate will likely continue to improve. 

There are several disorders of innate immunity. One important component of the immune system is the innate immune system (macrophage, neutrophil, dendritic cell, and complement system), which acts as the body's first line of defense against pathogens. However, there are certain disorders known as immunodeficiency disorders that can impair the functioning of this crucial defense mechanism [46]. Patients with innate immune deficiency disorders can begin to show symptoms at any age and frequently have infections that are unique or challenging to treat [47]. 

Phagocytic defects

Phagocytic defects, a subset of PIDs, underline a group of conditions impairing the critical immune process of phagocytosis [48]. Phagocytosis is the mechanism through which immune cells, such as neutrophils and macrophages, engulf and digest foreign particles, pathogens, and cellular debris. Dysregulation of this process can lead to compromised immune responses, recurrent infections, and inflammatory disorders [49].

Following are the types of primary Immunodeficiency with phagocytic defects.

Chronic Granulomatous Disease

Chronic granulomatous disease (CGD), a rare PID, owes its origin to hereditary factors. Mostly following an X-linked recessive pattern, CGD emerges from mutations within the CYBB gene, responsible for the crucial gp91phox subunit of the NADPH oxidase complex [50]. Autosomal recessive forms arise from mutations in CYBA, NCF1, and NCF2 genes, pivotal components of the NADPH oxidase complex [51,52]. This inheritance insight underscores the importance of a comprehensive approach involving genetic counseling and testing to ensure precise diagnosis and informed family planning [53].

The core of CGD's development lies in the malfunctioning NADPH oxidase complex, a critical generator of reactive oxygen species, pivotal for phagocyte-mediated microbe elimination [54]. Diminished NADPH oxidase activity leads to compromised phagocytic prowess and the inability to eradicate pathogens. As a result, CGD subjects experience recurrent bacterial and fungal infections, occasionally leading to granuloma formation. The body's hyperinflammatory response attempts to compensate for the flawed phagocytosis, manifesting as an accumulation of immune cells and cytokines. The understanding of these molecular intricacies has paved the way for targeted therapeutic interventions [55]. 

Clinical presentations of CGD unravel as heightened vulnerability to severe, recurring bacterial and fungal infections [56]. Organs such as the lungs, skin, lymph nodes, and liver often bear the brunt of these infections. Occasionally, granulomas develop, affecting multiple organ systems and yielding complications such as obstructive uropathy and gastrointestinal blockages. The disease's chronic nature and the diverse array of symptoms can often delay diagnosis, emphasizing the need for vigilant medical awareness, particularly in cases of recurrent infections and atypical clinical displays [57]. 

Recent diagnostic headway, encompassing genetic testing and flow cytometry, has drastically enhanced the precision and timeliness of CGD diagnoses [58]. Genetic testing offers pinpoint identification of mutations, enabling tailored therapeutic approaches. CGD management entails a multidisciplinary strategy encompassing antimicrobial prophylaxis, swift infection intervention, and occasionally, HSCT [59]. Emerging treatments like gene therapy hold promise for providing a definitive cure by addressing the genetic defect. Furthermore, therapies targeting enhanced neutrophil function are poised to enhance the quality of life for CGD patients [60]. 

Leukocyte Adhesion Deficiency

Leukocyte adhesion deficiency (LAD) is characterized by defects in the expression or function of adhesion molecules on leukocytes, resulting in impaired recruitment of phagocytes to the site of infection. This leads to severe bacterial infections and delayed wound healing [61]. LAD is a rare primary immune deficiency disorder inherited through an autosomal recessive pattern. To develop LAD, an individual must inherit two nonfunctional copies of the ITGB2 gene, which encodes CD18, a crucial subunit of β2 integrins. These genetic mutations hinder leukocyte adhesion molecule function, specifically the β2 integrin family, disrupting the capability of leukocytes, primarily neutrophils, to migrate, adhere, and phagocytize. As a result, the immune response against infections is compromised [62]. 

The root cause of LAD lies in malfunctioning leukocyte adhesion and migration processes. The defective binding of β2 integrins prevents leukocytes from attaching to endothelial cells, leading to a lack of mobilization to infection and inflammation sites. Consequently, individuals with LAD experience recurring bacterial and fungal infections, delayed healing of wounds, and prolonged inflammatory responses. Additionally, impaired neutrophil phagocytosis contributes to the heightened susceptibility to infections. The disruption of downstream cell signaling pathways triggered by β2 integrin binding further complicates immune responses [63].

LAD's clinical manifestations involve frequent and severe bacterial and fungal infections that impact various organs like the skin, respiratory tract, and gastrointestinal system. These infections pose life-threatening risks and are often unresponsive to standard treatments due to the compromised immune response. Furthermore, delayed wound healing, persistent skin infections, and periodontitis are common symptoms. An elevated white blood cell count might be observed as neutrophils are unable to migrate toward infection sites. Early recognition of these clinical signs is vital for prompt diagnosis and intervention [64].

Progress in diagnostic methods has facilitated earlier and more precise detection of LAD cases. Flow cytometry is pivotal for evaluating leukocyte adhesion molecule functionality and expression. Genetic testing is employed to confirm ITGB2 mutations, providing a definitive diagnosis. Managing LAD involves a comprehensive approach, incorporating prophylactic antibiotics, antimicrobial medications, and supportive care during infections. In severe cases, HSCT is considered a potential curative option, involving the replacement of defective cells with healthy ones. Promisingly, gene therapy is emerging as a potential solution to reinstate functional leukocyte adhesion molecules [65]. 

Complement defects

The complement system, an integral part of both innate and adaptive immunity, assumes a vital role in safeguarding the host against pathogens and contributing to debris removal. Recent strides in comprehending issues related to complement defects have yielded insights into disease mechanisms and novel avenues for therapeutic intervention [66].

The complement system encompasses an array of proteins and factors that initiate three pathways, the classical, lectin, and alternative pathways, culminating in the creation of C3 convertase, C5 convertase, and the formation of the membrane attack complex (MAC). These pathways converge at C3 convertase, facilitating opsonization, inflammation, and direct dissolution of pathogens [67]. Complement deficiencies make up fewer than 1% of all PIDs that have been found. Patients with these illnesses frequently present with serious or recurrent infections with encapsulated organisms or with a systemic autoimmune disease that resembles lupus erythematosus [47]. 

Classification of Complement Defects

Categorizing disorders linked to complement defects involves a wide spectrum of conditions, each stemming from disruptions within distinct components of the complement system. These conditions can be broadly classified into deficiencies of specific components, disturbances in complement activation regulation, and the presence of autoantibodies against complement components. 

Deficiencies of Specific Components

Deficits in certain complement components, including C1, C2, C3, and C4, lead to impaired activation of the complement cascade, resulting in compromised opsonization, inflammation, and immune complex clearance. These deficiencies render individuals more susceptible to infections and autoimmune disorders. For instance, insufficiency in C3, a central component in complement activation, gives rise to recurrent bacterial infections and heightened vulnerability to encapsulated bacteria like *Streptococcus pneumoniae* and *Haemophilus influenzae* [68]. 

Dysregulation of Complement Activation

Aberrant activation of the complement cascade results in excessive and uncontrolled complement activation, contributing to a range of autoimmune and inflammatory disorders. This dysregulation often arises due to mutations in complement regulatory proteins such as factor H, factor I, and membrane cofactor protein (MCP). Mutations in factor H, a pivotal regulator of the alternative pathway, are associated with conditions like atypical hemolytic uremic syndrome (aHUS) and age-related macular degeneration (AMD), where uncontrolled complement activation leads to tissue damage and inflammation [69]. 

Autoantibodies Against Complement Components

Autoantibodies targeting various complement components, such as C1q, C3, and C4, activate the complement system even in the absence of infection. This phenomenon leads to tissue damage, inflammation, and the development of autoimmune ailments. Autoantibodies against C1q are associated with systemic lupus erythematosus (SLE), a chronic autoimmune disorder characterized by autoantibody production and immune complex deposition [70]. 

Terminal Pathway Defects

Shortages in terminal pathway components, including C5, C6, C7, C8, and C9, impede the formation of the MAC, leading to compromised bacterial clearance and heightened susceptibility to Neisseria infections. C5 deficiencies result in the inability to generate the MAC, elevating the risk of recurring *Neisseria meningitidis* infections and a rare condition known as recurrent meningococcal disease [71]. 

Combined Deficiencies

Some complement-related disorders involve combined deficits of multiple complement components. These conditions often manifest complex clinical phenotypes and necessitate specialized diagnosis and management. Combined deficiencies of C1, C4, and C2 give rise to hereditary angioedema (HAE), an uncommon autosomal dominant disorder marked by recurrent episodes of severe, non-pitting edema in various body parts [72]. 

Disorders of immune dysregulation

Hemophagocytic Lymphohistiocytosis

Hemophagocytic lymphohistiocytosis (HLH) is an uncommon, potentially lethal illness characterized by the dysregulation of cytotoxic T lymphocytes, NK cells, and macrophages, resulting in hypercytokinemia and immune-mediated damage to many organ systems [73].

HLH has typically been divided into two categories: primary and secondary. Familial HLH (F-HLH) is another name for primary HLH. F-HLH is caused by homozygous or compound heterozygous genetic mutations that result in disruptive alterations that completely abolish the activity of cytotoxic T- and NK cells [73]. Secondary or acquired HLH develops as a result of extrinsic stimuli such as infection, cancer, rheumatic disorders, postallogeneic HSCT, medication hypersensitivity, or other underlying reasons. The Epstein-Barr virus (EBV) is the most prevalent infectious agent linked with HLH. HLH caused by EBV infection is more common in children and adolescents who have gene alterations linked to F-HLH and primary immunological illnesses such as X-linked lymphoproliferative syndromes type 1 and 2 [74]. Diagnostic criteria for HLH 2004 are shown in Table 1 [75].

**Table 1:** HLH diagnostic criteria 2004 Adapted form " Henter JI, Horne A, Aricó M, Egeler RM, Filipovich AH, Imashuku S, Ladisch S, McClain K, Webb D, Winiarski J, Janka G. HLH-2004: Diagnostic and therapeutic guidelines for hemophagocytic lymphohistiocytosis. Pediatr Blood Cancer. 2007 Feb;48(2):124-31. doi: 10.1002/pbc.21039. PMID: 16937360." HLH, hemophagocytic lymphohistiocytosis; NK, natural killer

The diagnosis of HLH requires that either one or two below are fulfilled:
A molecular diagnosis consistent with HLH
Diagnostic criteria for HLH fulfilled (five out of the eight criteria below)
Initial diagnostic criteria
Fever
Splenomegaly
Cytopenias (affecting ≥2 of 3 lineages in the peripheral blood): Hemoglobin <90 g/L (in infants <4 weeks: hemoglobin <100 g/L), platelets <100×10^9^/L, neutrophils <1.0×10^9^/L
Hypertriglyceridemia and/or hypofibrinogenemia: Fasting triglycerides ≥3.0 mmol/L (i.e., ≥265 mg/dL), fibrinogen ≤1.5 g/L
Hemophagocytosis in bone marrow or spleen or lymph nodes
New diagnostic criteria:
Low or absent NK-cell activity
Ferritin ≥500 mg/L
Soluble CD25 (i.e., soluble IL-2 receptor) ≥2400 U/mL

Autoimmune Lymphoproliferative Syndrome

The primary cause of autoimmune lymphoproliferative syndrome (ALPS), a condition marked by immunological dysregulation brought on by disturbed lymphocyte homeostasis, is mutations in the FAS-mediated apoptotic pathway. In a rare number of individuals with ALPS or ALPS-related illnesses, additional mutations of the genes for FAS-ligand (FASLG), Caspase 10 (CASP10), Caspase 8 (CASP8), NRAS, and KRAS have also been found [76]. ALPS revised diagnostic criteria 2009 are shown in Table 2 [77].

**Table 2:** ALPS revised diagnostic criteria 2009 Adapted from "Oliveira JB, Bleesing JJ, Dianzani U, Fleisher TA, Jaffe ES, Lenardo MJ, Rieux-Laucat F, Siegel RM, Su HC, Teachey DT, Rao VK. Revised diagnostic criteria and classification for the autoimmune lymphoproliferative syndrome (ALPS): report from the 2009 NIH International Workshop. Blood. 2010 Oct 7;116(14):e35-40. doi: 10.1182/blood-2010-04-280347. Epub 2010 Jun 10. PMID: 20538792; PMCID: PMC2953894." ALPS, autoimmune lymphoproliferative syndrome

The presence of both the required criteria plus one primary accessory criterion constitutes a conclusive diagnosis. The existence of both required requirements as well as one secondary accessory criterion leads to a likely diagnosis.
Required
Chronic (>6 months), nonmalignant, noninfectious lymphadenopathy or splenomegaly or both
Elevated CD3+TCRαβ+CD4−CD8−DNT cells (≥1.5% of total lymphocytes or 2.5% of CD3+lymphocytes) in the setting of normal or elevated lymphocyte counts
Accessory: primary and secondary
Primary
Defective lymphocyte apoptosis (in two separate assays)
Somatic or germline pathogenic mutation in FAS, FASLG, or CASP10
Secondary
Elevated plasma sFASL levels (>200 pg/mL) OR elevated plasma interleukin-10 levels (>20 pg/mL) OR elevated serum or plasma vitamin B12 levels (>1500 ng/L) OR elevated plasma interleukin-18 levels >500 pg/mL
Typical immunohistological findings as reviewed by an experienced hematopathologist
Autoimmune cytopenias (hemolytic anemia, thrombocytopenia, or neutropenia) AND elevated immunoglobulin G levels (polyclonal hypergammaglobulinemia)
Family history of a nonmalignant/noninfectious lymphoproliferation with or without autoimmunity

Immune Dysregulation, Polyendocrinopathy, Enteropathy, and X-linked Syndrome

IPEX syndrome, also known as immune dysregulation, polyendocrinopathy, enteropathy, and X-linked (IPEX) syndrome, is a rare monogenic autoimmune illness with a variety of clinical presentations, including late-onset or unusual symptoms as well as enteropathy, eczema, and type 1 diabetes. The mutation of the FOXP3 gene, which encodes a transcription factor necessary for the maintenance of Tregs, is the defining characteristic of IPEX notwithstanding the clinical variability. Tregs may exist in IPEX patients, but they may be unstable and functionally compromised, unable to stop the growth and cytokine production of effector T-cells [78]. With the help of typical clinical signs and the discovery of a hemizygous pathogenic mutation in FOXP3, the diagnosis of a male proband is made. Female carriers of a pathogenic mutation do not exhibit clinical symptoms, and no affected females have been identified [79].

Only a bone marrow transplant (BMT) gives hope for treating IPEX syndrome. Improved autoimmune resolution occurs when there is minimal organ damage, especially compared to non-transplanted persons receiving long-term immunosuppressive medication. First-line therapies include granulocyte colony-stimulating factor (G-CSF) for autoimmune neutropenia, rituximab for pemphigus nodularis and other autoantibody-mediated diseases, nutritional support, standard care for diabetes mellitus and autoimmune thyroid disease, and topical therapies for dermatitis [79].

Autoimmunity Polyendocrinopathy Candidiasis-Ectodermal Dystrophy

AIRE (autoimmune regulator) gene mutations lead to the uncommon recessively inherited condition known as autoimmunity polyendocrinopathy candidiasis-ectodermal dystrophy (APECED). Addison's disease, hypoparathyroidism, and type 1 diabetes are a few of the endocrine autoimmune illnesses that make up APECED. When fused to a heterologous DNA binding domain, the AIRE protein may initiate transcription of a reporter gene and has patterns that are indicative of a transcription regulator [80].

Diagnosis of primary immunodeficiency disorders

Common infections (such as sinusitis or otitis) that are often recurring, exceptionally prolonged, or severe are pathologic markers of PID. Sinusitis is by far the most typical illness in people with CVID [81]. Immunodeficiency testing may be necessary in cases of infections brought on by microbes that seldom cause illness in healthy individuals (such as Pneumocystis, Serratia, and nontuberculous mycobacteria) [82].

PID should be recognized when a typically minor pediatric illness results in a serious, potentially fatal complication, such as mastoiditis following otitis, a brain abscess connected to sinusitis, or pneumonia with empyema. Investigations should be conducted if neutrophil or lymphocyte counts are consistently low or high [82]. The Jeffrey Modell Foundation's Primary Immunodeficiency Resource Center provides a list of 10 indicators that a patient may have PID [83]. Those are 1) ≥8 new ear infections within one year, 2) ≥2 serious sinus infections within one year, 3) ≥2 months on antibiotics with little effect, 4) ≥2 episodes of pneumonia within one year, 5) failure of an infant to gain weight or grow normally, 6) recurrent deep skin or organ abscesses, 7) persistent thrush in the mouth or on the skin after age one year, 8) need IV antibiotics to clear infections, 9) ≥2 deep-seated infections, and 10) family history of PID [83].

A history of chronic antibiotic use or the appearance of two or more warning symptoms should trigger a PID examination [84]. Excessive inflammatory responses and autoimmunity, particularly cytopenias, are additional crucial PID symptoms. Additionally, it would be crucial for the immunologist to look into any secondary immunodeficiency reasons, such as drugs, other infections, immunoglobulin loss, and cancer [85,86,87]. A complete blood count (CBC) and blood smear are frequently the first steps in the immunologist's thorough immunological examination. These examinations are performed to determine whether lymphopenia, atypical or aberrant lymphocytes or phagocytic cells, and any related gross hematologic abnormalities that may be suggestive of PIDs are present. Considerable lymphopenia may be the initial sign of cellular T-cell immunodeficiency [47]. Lymphocyte proliferation tests and flow cytometry are additional crucial diagnostic techniques that enable the counting of B-cells, T-cells, and NK cells, as well as the analysis of lymphocyte markers, T-cell variability, and adhesion receptors that may be linked to certain immunological abnormalities [47]. Most instances of SCID and many cases of CID have aberrant results from standard flow cytometry examination [88].

Serum IgG, IgA, IgM, and IgE levels are measured in the initial investigation of patients with suspected B-cell (antibody-deficiency) illnesses (take note that the measurement of IgD is not helpful for the diagnosis of PIDs). The presence of B-cell immunodeficiencies may be indicated by serum levels that are significantly below age-appropriate reference values. The measurement of serum-specific antibody titers (often IgG) in response to vaccination antigens is the best method for confirming a diagnosis of an antibody-deficiency condition because some individuals with these illnesses have normal or just mildly lowered immunoglobulin levels [47]. In this method, a patient is immunized with protein antigens (such as tetanus toxoid) and polysaccharide antigens (such as pneumococcus) before and after the vaccine is administered. Antibody responses to these antigens are often weak or nonexistent in PIDs [88].

The diagnosis of innate diseases can be confirmed by neutrophil function assays (such as the dihydrorhodamine 1,2,3 response (DHR)) and stimulation assays for cytokine responses. For instance, aberrant neutrophil oxidase activity frequently signals CGD. Complement investigations that look at the quantity and/or function of certain complement proteins are crucial for the proper identification of complement-deficient illnesses. Accredited laboratories with proven proficiency in these tests and expertise in PID investigations should conduct these studies [88,89]. Tests for genetic reasons are also a crucial part of diagnosis, and in certain situations, more sophisticated tests for identifying the presence or function of cellular proteins may be utilized to confirm a diagnosis of PID [90]. Once the diagnosis is made, starting treatment as soon as possible is crucial since delays can result in irreversible organ damage or even death from an intense infection [89]. 

 

Treatment

PIDs are difficult to treat and typically require supportive and curative methods. As a result, the treatment should be managed by an immunologist with experience in treating these conditions [89,91].

Antibiotic Therapy

Depending on the kind of immunological malfunction present, infections are the most frequent forms of presentation in individuals with inborn errors of immunity (IEI). Inborn immune system defects make it difficult to treat infections in individuals, frequently necessitating the use of long-term, broad-spectrum drugs. A greater effort must be made to precisely identify infections because of the increased vulnerability to atypical agents, including the culture of afflicted tissues and genetic methods to identify the pathogen [92].

To reduce the frequency and severity of infections, particularly sinopulmonary infections brought on by common bacteria, prophylactic antibiotic therapy is widely used in the treatment of patients with PIDs. In some PIDs with more specialized susceptibilities, prophylactic antiviral and/or antifungal therapy may be necessary [93]. Recent research on the prophylactic use of azithromycin in immunoglobulin replacement therapy patients with antibody defects CVID and agammaglobulinemia revealed a decrease in the number of annual exacerbations, the need for antibiotics for treatment, and the risk of hospitalization [94].

Despite the lack of evidence to support the use of antibiotics in this population, isolated antibiotic prophylaxis is frequently given to patients with moderate hypogammaglobulinemia, selective IgA deficiency, or deficiency of IgG subclasses, which are not receiving immunoglobulin. Depending on the specific analysis of each case, the drugs may be used constantly or only during specific seasons of the year (particularly the winter). It is crucial to monitor these individuals' side effects and infection rates closely [95]. Examples of antibiotic prophylaxis regimens used in patients with immunodeficiency are *Pneumocystis jirovecii*: sulfamethoxazole-trimethoprim (SXT-TMP), infants >4 weeks of age and children, 5 mg/kg/day divided into two doses 3x/week (based on TMP, maximum 160 mg per day). Adults and adolescents with normal kidney function: based on TMP 80 mg/day or 160 mg daily or 160 mg 3x/week [96]. Staphylococcus spp, Gram-negative spp: SXT-TMP. Infants >4 weeks of age and children: 5 mg/kg/day divided into two daily doses (based on TMP, maximum 160 mg per day). Adults and adolescents: based on TMP 160 mg daily [96].

Immunoglobulin Replacement Therapy

Because antibody production is lacking or insufficient in around 50% to 75% of IEI patients, immunoglobulin replacement is necessary. Consequently, it is important to keep in mind the rise in secondary immunodeficiency cases that impair antibody production along with improvements in the treatment of lymphomas, leukemias, and other cancers [96]. Regarding the therapeutic benefit, immunoglobulin replacement therapy indications can be classified as 1) Proven benefit: immune system defects that affect B-cells, hypogammaglobulinemia, and inefficient antibody production; 2) likely benefit: immunoglobulins with apparently normal levels but with a qualitative defect in the specific antibody production; and 3) no benefit/contraindicated: selective IgA deficiency and IgG4 deficiency [96]. The main immunodeficiencies that require immunoglobulin replacement therapy are:

Agammaglobulinemia: A flaw in the development of B lymphocytes causes them to disappear, which prevents the synthesis of antibodies. The kappa-deleting element recombination circle (KREC) test can identify this category of illnesses during newborn screening, and full lymphocyte immunophenotyping can confirm the diagnosis later [96].

Hypogammaglobulinemia: In this instance, serum levels of immunoglobulins are lower, and antibody synthesis is decreased [97]. The most well-known example of this family of illnesses is CVID, which a number of genetic changes can cause [96].

Hyper IgM syndrome: IgM levels might be normal or increased, but IgA and IgG levels are decreased in certain disorders. Even if the B lymphocyte count is often normal, individuals often experience recurrent infections, which is a sign of agammaglobulinemia or mixed immunodeficiency [98].

Hemopoietic Stem Cell Transplantation

The replacement of these genetically changed cells with hematopoietic stem cells from healthy donors, more commonly known as bone marrow transplantation, is a highly logical treatment strategy because the majority of primary immunodeficiencies are caused by genetic flaws inherent to hematopoietic cells [99]. Patients with SCID and WAS had the first HSCT in patients with PID more than 50 years ago [100]. With more possible donor sources, better targeting of preparatory chemotherapy regimens, and improved support care, the strategy for HSCT and the overall risk have altered significantly over the past 20 years [101].

For the majority of cases of SCID, no chemotherapeutic conditioning is necessary when a completely HLA-matched sibling donor is utilized for stem cell transplantation (SCT). Additionally, using a matched sibling donor is linked to better survival rates, fewer issues such as graft versus host disease (GVHD), and more thorough B-cell engraftment [102]. Due to the fact that the majority of patients do not have HLA identical siblings, additional donor sources must be used, even if they may have a higher risk of graft rejection and morbidity [103]. Mismatched related donors (MMRD) are a potential source of donors and are frequently employed in particular series. At centers with expertise and ongoing improvement, success rates for these transplants are greater [104]. Additionally utilized are umbilical cord blood (UCB) and matched unrelated donors (MUD). Because there are only a finite amount of donor cells in the umbilical cord, UCB transplantation is typically insufficient for bigger patients [103]. Most notably, when newborns receive SCT at 3.5 months of age, there is an improvement in survival. If there is no sign of active infection after a transplant in a child with SCID, they may recover successfully [103]. However, age at transplant is a crucial issue since younger patients with infections recover more quickly than older patients with infections [105]. This emphasizes the value of early diagnosis by newborn metabolic screening (NBS) to enhance the results for SCID patients [103].

CGD, a PID that frequently results in profound infections and inflammatory problems in several organs, can also be cured by HSCT. Significant pre-transplant conditioning is necessary, and the recipient frequently has comorbidities that might affect the development of post-transplant problems [106]. A multicenter research detailing transplantation utilizing reduced-intensity conditioning and matched unrelated or sibling donors demonstrated how successful HSCT for CGD has become in recent years [107]. SCT can also be used to treat other immunodeficiencies, such as WAS effectively, X-linked high IgM syndrome, and X-linked lymphoproliferative illness [108]. New methods for preparing the graft and training the host may enable effective SCT while almost reducing toxicity [103]. Using an anti-CD45 antibody is a potential non-chemotherapy conditioning technique since CD45 is found on the surface of all nucleated hematopoietic cells and their progenitors [109,110].

Additionally, strategies have been developed to manage severe viral infection, a post-transplant consequence that is frequently fatal. In the post-transplant period, viruses, including cytomegalovirus (CMV), EBV, and adenovirus, among others, can be lethal and can be very difficult to manage with standard antiviral therapies alone [103]. Donor lymphocyte infusion (DLI) is one immunologic form of therapy, although this strategy has limitations due to a lack of specificity against the virus and danger for GVHD [109].

## Conclusions

Immunodeficiency has always been a mystery and a potential research zone for many years. It is unique how each of our immune systems is so similar in composition and yet different in function, and how some individuals are more prone to infections when compared to others with the same genetic composition of the immune system. This article has given a detailed review of the common and uncommon PIDs and the emerging management options, which will provide insights and open up many doors to further the knowledge and understanding of these subsets. It is of prime importance to have a glimpse of these disorders to correctly diagnose them, as in most cases time is of the essence to start the therapy, which can alter the outcome significantly in these immunodeficiency cases.
